# The Non-Canonical CTD of RNAP-II Is Essential for Productive RNA Synthesis in *Trypanosoma brucei*

**DOI:** 10.1371/journal.pone.0006959

**Published:** 2009-09-09

**Authors:** Anish Das, Vivian Bellofatto

**Affiliations:** Department of Microbiology and Molecular Genetics, University of Medicine and Dentistry-New Jersey Medical School, Newark, New Jersey, United States of America; BMSI-A*STAR, Singapore

## Abstract

The carboxy-terminal domain (CTD) of the largest subunit (RPB1) of RNA polymerase II (RNAP-II) is essential for gene expression in metazoa and yeast. The canonical CTD is characterized by heptapeptide repeats. Differential phosphorylation of canonical CTD orchestrates transcriptional and co-transcriptional maturation of mRNA and snRNA. Many organisms, including trypanosomes, lack a canonical CTD. In these organisms, the CTD is called a non-canonical CTD or pseudo-CTD (ΨCTD. In the African trypanosome, *Trypanosoma brucei*, the ΨCTD is ∼285 amino acids long, rich in serines and prolines, and phosphorylated. We report that *T. brucei* RNAP-II lacking the entire ΨCTD or containing only a 95-amino-acid-long ΨCTD failed to support cell viability. In contrast, RNAP-II with a 186-amino-acid-long ΨCTD maintained cellular growth. RNAP-II with ΨCTD truncations resulted in abortive initiation of transcription. These data establish that non-canonical CTDs play an important role in gene expression.

## Introduction

RNA polymerase II (RNAP-II) is the eukaryotic enzyme responsible for synthesis of mRNA. This enzyme is an ∼550 kDa complex consisting of twelve subunits, RPB1-12 [1]. The carboxy-terminal domain (CTD) of the largest subunit, RPB1, is essential for cell survival. In well-studied organisms, such as metazoa and yeast, the CTD consists of consensus heptapeptide repeats having the sequence Y_1_S_2_P_3_T_4_S_5_P_6_S_7_ ([2], [3] and reviewed in [4], [5]). The CTD plays a pivotal role in RNA production by recruiting multiple proteins that modulate transcription initiation, transcription elongation, transcription termination, co-transcriptional mRNA 5′ capping, RNA splicing, and RNA polyadenylation [6]–[8]. Interaction of proteins with the CTD is orchestrated by dynamic and differential phosphorylation. The patterns of phosphorylation make up the “CTD code” [5], [9]. Non-canonical CTDs, which lack the heptapeptide repeats and thus are pseudo CTDs (ΨCTDs), exist in a wide variety of eukaryotic organisms [10], [11]. For example, the early branching protozoan, *Trichomonas vaginalis*, and the multicellular red alga, *Bonnemaisonia hamifera*, as well as members of the Apicomplexian, Stylonichia, and Kinetoplastid groups contain ΨCTDs as part of their RNAP-II machinery [12], [13]. We sought to determine whether a ΨCTD is essential for gene expression.

**Figure 1:**
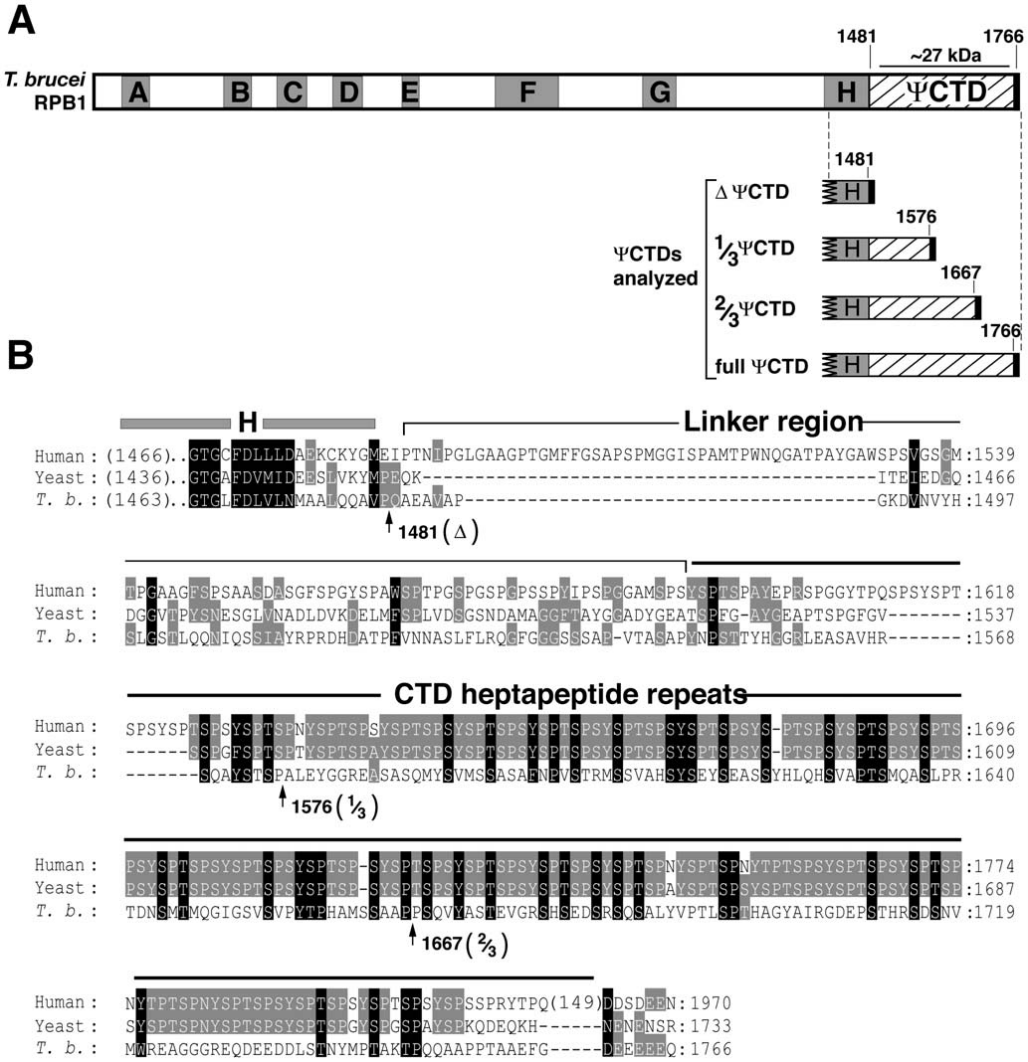
The ΨCTD of *T. brucei* RNAP-II lacks conserved heptapeptide sequence motifs found in most other eukaryotes. (A) Domain structure of *T. brucei* RPB1 showing the presence of the characteristic A–H domains and the divergent carboxy-terminal domain. The carboxy terminus of *T. brucei* RPB1 lacks the highly conserved YSPTSPS repeats that orchestrate multiple co-transcriptional processes in the well-studied eukarya. Four different RPB1 proteins analyzed in this study are diagrammed: RPB1 containing no ΨCTD (ΔΨCTD, truncated at amino acid 1481), one-third of ΨCTD (1/3 ΨCTD, truncated at amino acid 1576), two-thirds of ΨCTD (2/3 ΨCTD, truncated at amino acid 1667), and full length ΨCTD (full ΨCTD). The heavy line at the C-terminus of each truncation represents the five conserved amino acids (E/DEEEQ). The location of the ∼27 kDa Lys-C fragment is indicated with a horizontal line over the ΨCTD. (B) Alignment of the human and yeast CTD with the ΨCTD sequence of *T. brucei* RPB1. A portion of the H domain is included (gray bar) as reference. The CTD's linker region (thin line) and the heptapeptide repeat domain (thick line) are shown. Thirty-one of the 52 heptapeptide repeats in the mammalian CTD are shown. The ΨCTD truncations are defined by arrows following the final amino acid contained in each mutant protein. Grey boxes show similar amino acids in two sequences, black boxes show similar amino acids in all three sequences.

Among the Kinetoplastids, the largest subunit of RNAP-II has an ∼300 amino acid-long ΨCTD that lacks heptapeptide repeats and exhibits little amino acid similarity to CTDs of other organisms [14]–[17]. In the African trypanosome, *Trypanosoma brucei*, the ΨCTD of RNAP-II is an ∼285 amino acid-long carboxyl terminal extension of RPB1 (Figure 1 and [15], [16]). In *T. brucei*, RNAP-II synthesizes two very different types of RNA, a long polycistronic precursor mRNA (pre-mRNA) and a short, capped Spliced Leader (SL) RNA. mRNA maturation require addition of SL RNA to pre-mRNAs through a trans-splicing reaction. Different forms of RNAP-II, associated with specific transcription factors, likely transcribe SL RNAs and pre-mRNAs. Indeed the *T. brucei* homolog of the yeast transcription factor Spt16 appears to associate with protein-coding genes but not with the SL RNA gene [18]. In contrast, the *T. brucei* homolog of the mammalian snRNA transcription factor SNAPc appears to associate only with the SL RNA gene promoter, although one subunit of SNAPc in *Leishmania major*, a relate parasite, occupies regions upstream of protein-coding genes [19]. Whether RNAP-II ΨCTD is important for transcription of either the SL RNA gene and/or protein-coding genes is unknown.

To address if non-canonical CTDs play a fundamental role in gene expression, we undertook a study of *T. brucei* RNAP-II ΨCTD. We altered RNAP-II and tested its function using an *in vivo* assay system and discovered that the ΨCTD is essential for cell survivial and production of both SL RNA and mRNA. Nascent transcription analysis demonstrated that the ΨCTD is required specifically for productive transcription initiation, as an RNAP-II severely truncated within the ΨCTD caused abortive initiation. These results demonstrate that a non-canonical CTD is a vital component of the RNAP-II machinery in eukaryotic cells.

## Results

### *T. brucei* ΨCTD undergoes phosphorylation

**Figure 2:**
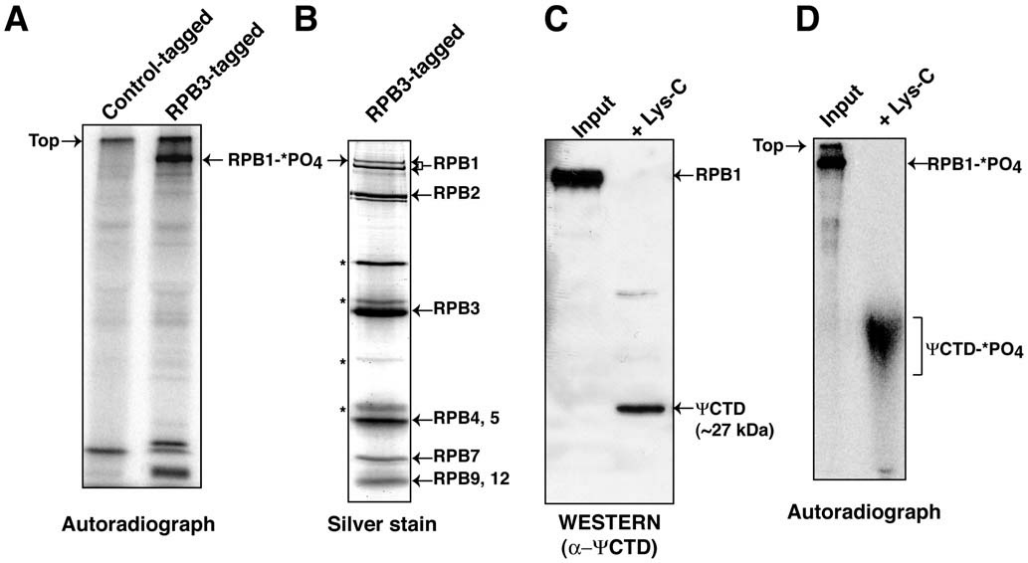
The ΨCTD of *T. brucei* RNAP-II undergoes phosphorylation. (A) RNAP-II tandem affinity purified from RPB3-tagged cells metabolically labeled with ^32^P-orthophosphate was separated on SDS-8% PAGE and autoradiographed. ^32^P-labeled RPB1 (RPB1-*PO_4_) is shown (right lane). The left lane contains a similar purification from A7-tagged cells and served as a control for protein purification[21]. (B) Eight of the twelve subunits of RNAP-II are shown on silver-stained SDS-8% PAGE. The two RPB1 bands were confirmed as the phosphorylated and non-phosphorylated forms of RPB1 (II0 and IIA) by the conversion of II0 to IIA after lambda protein phosphatase treatment. II0 and IIA were detected using anti-ΨCTD antibody (Daniels J-P, Wickstead, W and Gull, K, personal communication). Asterisks mark four yet to be identified polypeptides. (C) A Western blot using anti-ΨCTD antibodies identified the ∼27 kDa as in-gel digestion product of RPB1 with endoproteinase Lys-C. The input (left lane) was RPB1 prior to proteolysis. (D) An autoradiograph that contains the ΨCTD (ΨCTD -*PO4, right lane), generated by in-gel digestion of RPB1-*PO_4_ with Lys-C. The ΨCTD-*PO_4_ migrates more slowly than the unmodified peptide and is broad band probably because it contains a heterogeneous number of modifications. The input (left lane) was RPB1-*PO_4_ prior to proteolysis.

It has been shown previously that *T. brucei* RNAP-II is modified by phosphorylation, despite the lack of consensus heptapeptide repeat sequences [20]. To determine whether the phosphorylation occurs within the ΨCTD, we directly visualized RNAP-II labeled with ^32^P-orthophosphate. For this work, we tagged RNAP-II in a *T. brucei* procyclic cell line by stably-expressing the RPB3 subunit of the enzyme with a tandem affinity tag. These transgenic cells were metabolically labeled with ^32^P-orthophosphate and RNAP-II was purified from nuclear extracts using protein A and streptavidin-binding peptide affinity chromatography. An autoradiograph of the purified protein, separated into polypeptides by denaturing gel electrophoresis, indicated that the largest subunit (RPB1) is modified by phosphorylation (Figure 2A). A stained gel of the purified proteins revealed both phosphorylated and non-phosphorylated forms of RPB1 (Figure 2B). Seven of the eleven other RNAP-II subunits, as verified by mass spectrometry, were also seen [21]. To determine whether phosphorylation occurs in the ΨCTD of RPB1, ^32^P-labeled RPB1 was subjected to in-gel proteolysis by endoproteinase Lys-C, which cleaves the peptide bond at the carboxy side of lysine. Lys-C digestion of RPB1 generated a 253 amino acid long, ∼27 kDa, peptide consisting only of the ΨCTD (from amino acid 1491–1744) minus its terminal 21 amino acids (Figure 1A). The remainder of RPB1 was fragmented into <76 amino acid-long peptides. Immunoblotting with anti-ΨCTD antibodies detected the ∼27 kDa peptide (Figure 2C). Lys-C digestion products from ^32^P-orthophosphate-labeled RPB1 are shown in Figure 2D. The heterogeneous collection of radiolabeled peptides migrating more slowly than ∼27 kDa are ΨCTD peptides that differ in phosphorylation patterns. The immunoblot in Figure 2C shows most clearly the non-phosphorylated ΨCTD because the phosphorylated peptides migrate as a broad band that eludes antibody detection. Thus, our analysis demonstrates that although *T. brucei* RNAP-II ΨCTD lacks a heptapeptide repeat sequence, it undergoes phosphorylation.

### RNAi-targeting of mRNA 3′UTR allows regulated depletion of RPB1

**Figure 3:**
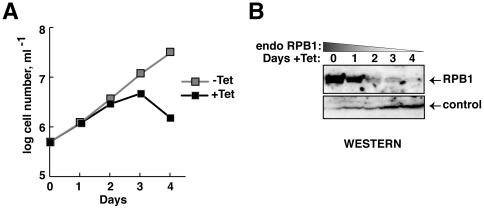
Depletion of RPB1 by RNAi causes lethality. (A) Growth curves of a clonal transgenic RNAi-containing cell line in the presence (black squares) and absence (gray squares) of tetracycline. Tetracycline was added on day 0. (B) Western analysis of whole cell lysates prepared on days 0–4 after RNAi induction, using anti-ΨCTD antibodies, shows depletion of *T. brucei* RPB1. Loading controls are in the lower panel.

To test directly the importance of RNAP-II ΨCTD in transcription, we used RNAi to deplete endogenous RNAP-II (referred to as ‘endo’) and replace it with mutant RNAP-II, conditionally expressed from an exogenously provided DNA cassette. Endo RNAP-II relies upon RPB1 that is encoded by two sets of allelic gene copies on chromosomes 4 and 8. These genes differ slightly in their protein-coding regions. Fortunately, RPB1 mRNAs generated from all four alleles contain identical 3′UTRs, as determined by analysis of RPB1 mRNA by 3′ RACE-PCR (data not shown). We generated cell lines containing inducible RNAi that targeted the RPB1 3′UTR. As expected, targeting 3′UTR of RPB1 mRNA by tetracycline-inducible RNAi expression stops cellular growth of procyclic *T. brucei* by day 2, in contrast to uninduced cells. The induced cells progressed to cell death by day 4 (Figure 3A). Western analysis of total cellular protein showed decrease in RPB1 beginning by day 1 (Figure 3B).

### RNAP-II containing 1/3 ΨCTD is toxic and causes cell death

To study whether RPB1 mutant proteins can support transcription, we expressed tagged versions of full, 2/3, 1/3 and null (Δ) ΨCTD domain-containing proteins (Figure 1A). The full ΨCTD or 2/3 ΨCTD RPB1 transgenic cell lines were readily established. However, cell lines with a 1/3 ΨCTD or Δ ΨCTD RPB1 were difficult to establish: only one cell line for each construct was obtained after multiple transfections. The 1/3 ΨCTD or Δ ΨCTD RPB1-containing parasites grew poorly and had low levels of non-phosphorylated tagged-RPB1 (Figure S1). These results indicated that expression of either the 1/3 ΨCTD or Δ ΨCTD poison cells and prohibit selection of robustly expressing cell lines.

**Figure 4:**
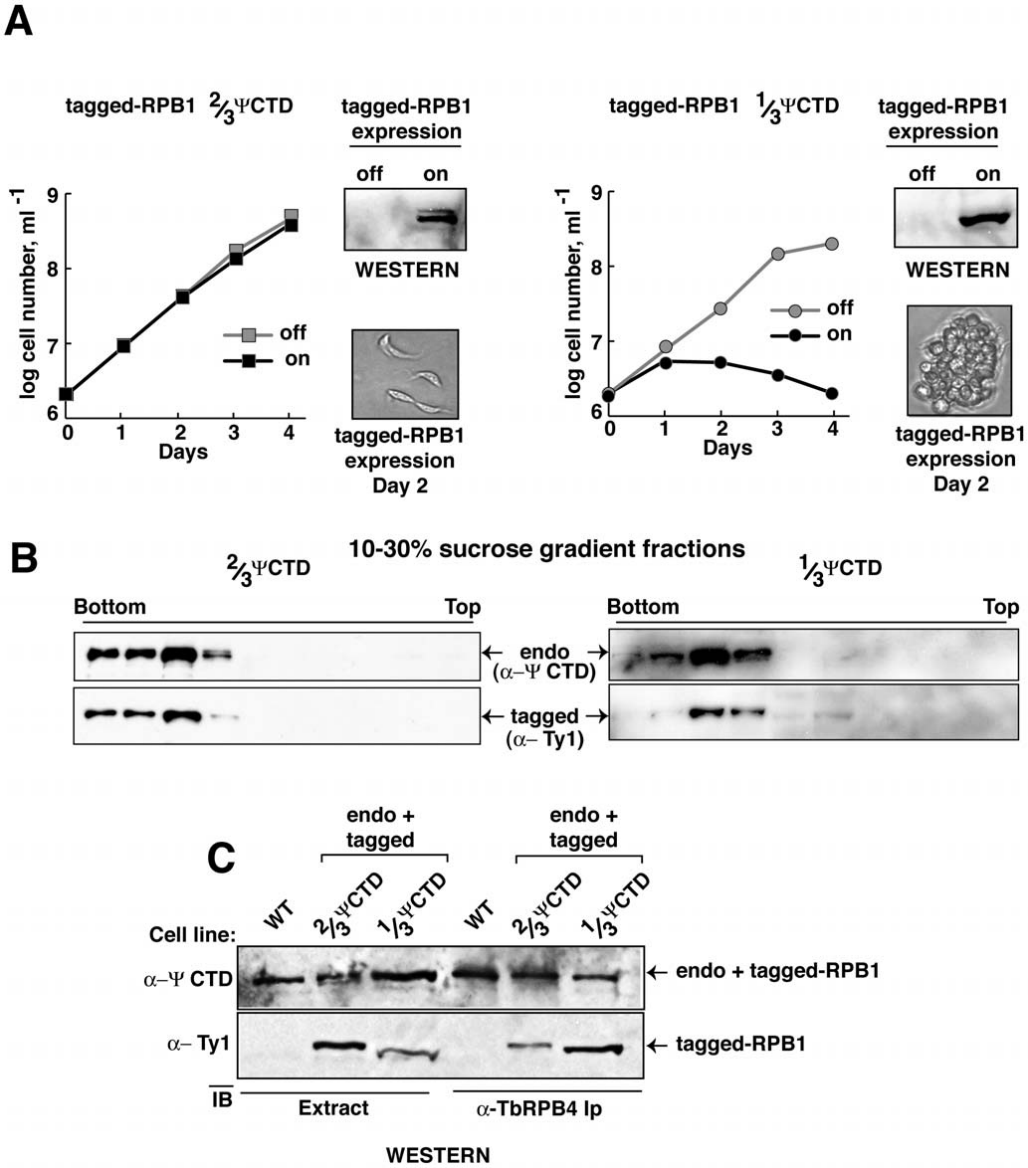
RNAP-II with a 1/3 ΨCTD poisons cell growth. (A) Growth curves of transgenic *T. brucei* cell lines in the presence (black, on) or absence (gray, off) of either 2/3 ΨCTD (left panel, square) or 1/3 ΨCTD RPB1 (right panel, circle). Western analyses of whole cell lysates before (off) and two days after (on) induction of exogenous RPB1 are shown. Microscopic images of cellular morphology two days post-induction of exogenous proteins are shown. (B) Western analyses of 10 to 30% sucrose gradient fractions. Both endogenous *T. brucei* RPB1 (top panel, endo) and exogenous RPB1 (bottom panel, tagged) are shown. (C) Immunoblot analysis of whole cell extract (left three lanes, Extract) or anti-*T. brucei* RPB4 immune-captured proteins (right three lanes, α-RPB4) from wild-type (WT) and transgenic parasites expressing exogenous 2/3 ΨCTD or 1/3 ΨCTD RPB1.

To investigate whether 1/3 ΨCTD poisons the RNAP-II enzyme, we tailored RPB1 transgenes to express in an inducible fashion (Table 1). The discovery that RNAP-II with 2/3 ΨCTD was well tolerated and modified by phosphorylation (Figure S1), whereas 1/3 ΨCTD was not, prompted us to compare 2/3 ΨCTD with 1/3 ΨCTD RNAP-II in our studies. Induced expression of 2/3 ΨCTD RNAP-II resulted in normal cellular growth (Figure 4A). However, induced expression of 1/3 ΨCTD RNAP-II was toxic to cells. The crippling effect of 1/3 ΨCTD RNAP-II was evident in the rapid growth arrest and loss of viable cells. Microscopic observation following expression of 1/3 ΨCTD RNAP-II revealed striking changes: cells became rounded and formed large aggregates. In contrast, cell morphology was normal following expression of RNAP-II with 2/3 ΨCTD (Figure 4A).

**Table 1:** RPB1 expression schemes for ΨCTD analysis.

	−Tet	+Tet	
Endogenous RPB1	On	On	Fig. 4
Inducible tagged-RPB1	Off	On	
Endogenous RPB1	On	Off	Fig. 5–7
Inducible tagged-RPB1	Off	On	

To verify that the 1/3 ΨCTD RPB1 was incorporated into RNAP-II, we analyzed enzyme complexes (Figure 4B). Fractionation of nuclear extract by sucrose density-gradient centrifugation confirmed that tagged-2/3 ΨCTD and tagged-1/3 ΨCTD RPB1 assembled into complexes that migrated near the bottom of the gradient. These same fractions were enriched for endogenous RNAP-II. Transcriptionally active endogenous RNAP-II also migrated at this position in similar sedimentation velocity measurements (data not shown). Western blot analysis of immunoprecipitated proteins captured by polyclonal anti-RPB4 antibody demonstrated the presence of tagged-1/3 ΨCTD, tagged-2/3 ΨCTD, or endogenous RPB1 in RNAP-II (Figure 4C). These results confirmed that 2/3 ΨCTD or 1/3 ΨCTD RPB1 were incorporated into RNAP-II. Thus, RNAP-II with 1/3 ΨCTD is toxic to cells and produces a dominant negative effect over endogenous RNAP-II.

### RNAP-II containing 2/3 ΨCTD rescues cellular growth in absence of endogenous ΨCTD

**Figure 5:**
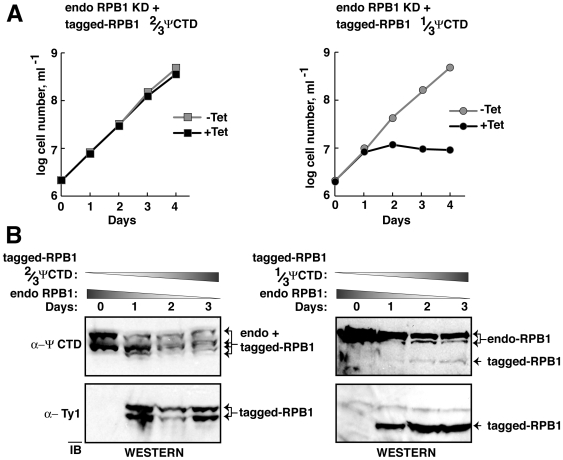
RNAP-II with a 2/3 ΨCTD supports cell growth, whereas enzyme with a 1/3 ΨCTD does not. (A) Growth curves of *T. brucei* RNAi cell lines in the presence (black squares and circles) or absence of tetracycline (gray squares and circles). Tetracycline causes RNAi-dependent knock down of endogenous RPB1 and induction of exogenous RPB1. 2/3 ΨCTD RPB1 is in the left panel, 1/3 ΨCTD RPB1 is in the right panel. (B) Immunoblot of whole cell lysates from cultures shown in (A) after tetracycline induction for 0–3 days. Endogenous and exogenous RPB1 were detected as in Figure 4. The four bands in the left upper portion are the phosphorylated and non-phosphorylated forms of the endogenous and 2/3 ΨCTD RBP1 proteins. The upper right panel displays three bands as the 1/3 ΨCTD RPB1 is not phosphorylated.

Although a 1/3 ΨCTD RNAP-II had a dominant-negative effect, cells expressing both full-length ΨCTD and 2/3 ΨCTD RNAP-II grew normally. We speculated that RNAP-II harboring a 2/3 ΨCTD might be as proficient as the endogenous enzyme in supporting cell growth. Thus, a 2/3 ΨCTD RNAP-II was tested for its ability to rescue cells depleted of endogenous RNAP-II (Figure 5). We established stable cell lines containing a regulated RNAi construct and a regulated tagged-RPB1 gene. Addition of tetracycline induced RNAi production and tagged RPB1 expression. Thus, endogenous RPB1 was selectively destroyed and the tagged-RPB1 proteins that contain either the 2/3 ΨCTD or 1/3 ΨCTD were produced. Growth curves showed that 2/3 ΨCTD RNAP-II sustained cellular growth after RNAi-depletion of endogenous RPB1 (Figure 5A). In contrast, 1/3 ΨCTD RNAP-II failed to rescue the cells.

Endogenous RPB1 depletion was monitored by immunoblotting with anti-ΨCTD antibody. The top, left blot in Figure 5B shows that both phosphorylated and non-phosphorylated forms of the endogenous RPB1 decreased after RNAi-targeting. In addition, both phosphorylated and non-phosphorylated forms of the tagged-2/3 ΨCTD RPB1 were present after induction. Similar results are shown in the top, right blot in Figure 5B, although the diminished number of epitopes on the 1/3 ΨCTD resulted in weak antibody detection. Expression of tagged proteins was independently monitored in immunoblots by using anti-tag antibodies (Figure 5B, lower blots). As noted above, the 1/3 ΨCTD RPB1 failed to undergo phosphorylation, and thus a single band corresponding to the tagged protein was observed. These data confirmed that induced expression of tagged 2/3 and 1/3 ΨCTD RPB1 occurred in our experiments. Taken together, these results argue that ΨCTD contributes to cell viability, likely as an essential component of functional RNAP-II.

### RNAP-II requires a 2/3 ΨCTD to maintain steady-state transcript levels

**Figure 6 pone-0006959-g006:**
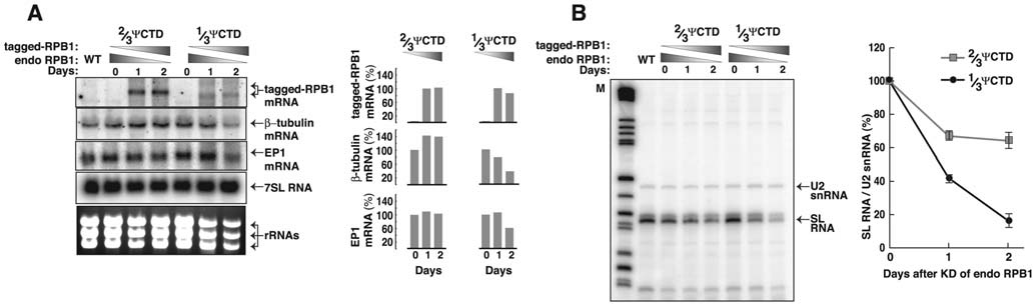
RNAP-II requires a 2/3 ΨCTD to maintain steady-state levels of SL RNA and mRNA. (A) Northern blot analysis of total RNA prepared from wild-type (WT) and transgenic *T. brucei* cell lines before (day 0) and after 1 and 2 days following RNAi-dependent knock down of endogenous *T. brucei* RPB1 and induction of exogenous 2/3 ΨCTD or 1/3 ΨCTD RPB1. Ribosomal RNAs (rRNAs) were visualized by ethidium bromide staining. Transgene-derived RPB1 mRNA, β-tubulin mRNA, EP1 mRNA, and 7SL RNA were visualized by hybridizing with specific radiolabelled oligonucleotides. β-tubulin and EP1 mRNA levels were normalized using 7SL RNA and considered 100% on day 0. Transgene-derived RPB1 mRNA is indicated as 100% on day 1. (B) SL RNA was analyzed by primer extension of RNA from cells expressing RPB1 as in (A). Radiolabelled oligonucleotides hybridized to either SL RNA or U2 snRNA in the primer extension. A line graph shows percent SL RNA with respect to U2 snRNA after two days of tetracycline induction. Day 0 is considered 100%. A representative experiment from three independent experiments is shown.

To determine the role of ΨCTD on RNAP-II transcription, first we analyzed transgenic cells for steady-state transcript levels following concomitant endogenous RPB1 depletion and tagged-RPB1 induction. Northern blot analyses of total RNA confirmed that expression of the tagged-RPB1 mRNA from transgenes containing either the 2/3 ΨCTD or 1/3 ΨCTD was tightly regulated and induced with tetracycline (Figure 6A). These data are shown schematically in the right portion of the panel. Levels of 7SL RNA, which requires RNAP-III, were unaffected during the two-day course of the experiment. In contrast, levels of β-tubulin mRNA, which requires RNAP-II for synthesis, were markedly reduced in cells expressing the 1/3 ΨCTD RNAP-II; only ∼40% of the β-tubulin mRNA remained by day 2 after induction. In cells containing 2/3 ΨCTD RNAP-II, no decrease in β-tubulin mRNA was observed. The differential effect on β-tubulin levels supported the observation that while RNAP-II with 1/3 ΨCTD was crippled, RNAP-II with 2/3 ΨCTD was functional. Finally, levels of procyclin/EP1 mRNA, which requires RNAP-I, indicated that ∼60% of the procyclin/EP1 mRNA was present in 1/3 ΨCTD-expressing cells in comparisons to cells expressing endogenous enzyme. The decrease in EP1 mRNA steady-state levels was likely due to inefficient mRNA maturation, which requires the RNAP-II-dependent SL RNA transcript.

To test this prediction, we assessed SL RNA levels by primer extension analysis (Figure 6B). Cells that relied on 1/3 ΨCTD RNAP-II exhibited a striking decrease in SL RNA steady-state levels; there was a 70% decrease on day 1 and a 85% decrease by day 2. In contrast, cells containing 2/3 ΨCTD RNAP-II maintained SL RNA levels. They experienced only a ∼30% decrease on day 1 that did not change by day 2. Thus, 1/3 ΨCTD RNAP-II was probably incapable of making new SL RNA transcripts and thus failed to maintain a reservoir of SL RNA necessary for efficient mRNA maturation.

### RNAP-II with 1/3 ΨCTD is defective in transcription and causes abortive initiation

**Figure 7:**
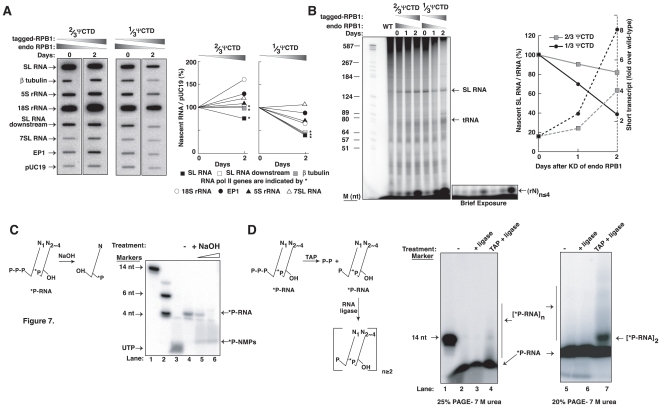
RNAP-II with 1/3 ΨCTD is impaired in synthesizing SL RNA and pre-mRNA and produced abortive initiation. (A) Analysis of nascent RNA prepared from cells, as in Figure 6, before (day 0) and 2 days after tetracycline induction. PCR-generated DNAs for the indicated genes were immobilized on nylon membrane and hybridized with radiolabelled nascent RNA prepared from permeabilized cells. A line diagram indicates the changes in nascent RNA, as measured by densitometric analysis of slots from two independent experiments, and normalized to the pUC19 hybridization signal. * marks RNAP-II-transcribed SL RNA (black square), β-tubulin (gray square) and the SL RNA downstream region (open square). RNAP-I-transcribed EP1 (black circle), 18S rRNA (open circle), and RNAP-III-transcribed 7SL RNA (open triangle) and 5S rRNA (black triangle), are also shown. Day 0 is indicated as 100%. (B) Nascent SL RNA, from wild-type (WT) and transgenic *T. brucei* cells before (day 0) and after 1 and 2 days of exogenous RPB1 expression as in (A) were analyzed by denaturing 8% PAGE. Labeled SL RNA and tRNA are shown. Increasing amounts of short transcripts, (rN)_n≤4_, are produced in permeabilized cells expressing the 1/3 ΨCTD RNAP-II. The short transcripts are evident in the brief exposure. Nascent SL RNA levels with respect to tRNA levels are indicated on the left y-axis. Day 0 is indicated as 100%. The increase in short transcripts before (day 0) and after 1 and 2 days after RPB1 expression is shown on the right y-axis. Fold increase was always compared to the wild-type level. Markers are shown in the leftmost lane of the gel. (C) Analysis of the short transcripts produced in permeabilized cells expressing 1/3 ΨCTD RNAP-II on day 2 of tetracycline induction. Radiolabelled mononucleotides were produced by alkali hydrolysis of the gel-purified short transcripts, identifying them as RNA. Markers (lanes 1 and 2) and ^32^P-UTP (lane 3) are shown on the gel. (D) The short transcripts, when treated with TAP followed by RNA ligase, formed larger RNAs by self-ligation (lanes 4 and 7). Larger RNAs were not detected in the absence of TAP enzyme (lanes 3 and 6), indicating the presence of a tri-phosphate group at the 5′ end of the short transcripts. Lanes 2 and 5 contain the input (eluted) RNA. Marker is shown in lane 1.

To examine how the 1/3 ΨCTD impairs RNAP-II function, we assessed nascent RNA synthesis in transgenic cells before and after expression of 1/3 ΨCTD RPB1-containing enzyme. In comparison to uninduced cells, cells utilizing 1/3 ΨCTD RNAP-II poorly synthesized β−tubulin and SL RNA (Figure 7A). Reduced synthesis of β−tubulin and SL RNA was also observed in cells utilizing 2/3 ΨCTD-containing RNAP-II, although this reduction was minimal. For the cells relying on the 1/3 ΨCTD RNAP-II, we observed an overall decrease in RNAP-I (procyclin/EP1 and 18S rRNA) and RNAP-III (7SL RNA and 5S rRNA) transcription, which probably indicates a general reduction in their transcriptional capacity.

We verified that the 1/3 ΨCTD RNAP-II is less efficient in transcription than its wild-type or 2/3 ΨCTD-containing homolog by direct visualization of newly synthesized, radiolabeled SL RNA (Figure 7B). Nascent SL RNA was significantly more reduced in cells containing 1/3 ΨCTD RNAP-II compared to cells containing 2/3 ΨCTD RNAP-II (∼60% compared to 20%), after two days of expression. Interestingly, we also observed an increase in small radiolabeled molecules migrating near the bottom of the gel, as shown in the brief exposure in Figure 7B. Compared to wild-type, cells expressing either 2/3 ΨCTD RNAP-II or 1/3 ΨCTD RNAP-II produced increased level of very short (∼4 nt long) transcripts. The amount of short transcripts was greater in cells containing 1/3 ΨCTD RNAP-II (eight-fold over wild-type) than in cells containing 2/3 ΨCTD (four-fold over wild-type). We postulate that these RNAs were generated from abortive initiation.

We first determined that the radiolabelled molecules were RNA, and thus sensitive to alkali treatment (Figure 7C). Mononucleotides were produced from gel-purified, short transcripts (see Figure 7B, brief exposure). To confirm that the short RNAs indeed originated from transcription initiation events, we characterized their 5′ end. Short transcripts, treated with Tobacco Acid Pyrophosphatase (TAP), were ligated to form a ladder of longer RNAs (Figure 7D). No ligation products were observed when TAP-treatment was omitted. Taken together, the nascent transcription experiments supported the conclusion that RNAP-II requires a ΨCTD to enter into a productive transcription process.

## Discussion

The present work describes the first characterization of an RNAP-II non-canonical CTD, which is found in many single- cell organisms. The *T. brucei* RNAP-II uses a non-canonical CTD for cell growth and viability. The growth defect associated with ΨCTD deletions was likely a direct result of decreased mRNA production. Specifically, when the ΨCTD is truncated, defective RNAP-II produced less mRNA and SL RNA and increased abortive initiation products.

A consequence of a non-canonical CTD is the opportunity for RNAP-II to partner with unknown proteins. This contention is best illuminated by the elegant biochemical and structural studies in *Trichomonas vaginalis*. Here, a non-canonical CTD partners with a *Trichomonas*-specific protein, IBP39, to stably assemble RNAP-II at the initiator element of promoters [12]. Affinity-purification of RNAP-II from *T. brucei* revealed several copurified proteins that appear to be trypanosome-specific [21], [22]. We speculate that some of these proteins participate in gene expression through specific interaction with ΨCTD. Indeed, a ten amino acid motif (SSYHLQ-SVAP) is shared among trypanosomes within the serine/proline-rich subdomain of ΨCTD (Figure 1). This motif is within the 2/3 ΨCTD truncation that rescued cells when wild-type RNAP-II was depleted (Figures 5 and 6). In summary, these observations are consistent with non-canonical CTD having significant roles in organism-specific RNAP-II machineries.

Modification of canonical CTD by phosphorylation at specific serine residues is required for orchestrating transcriptional activities in better studied eukaryotes. It is known that a S_5_ phospho CTD-code dictates that an enzyme leaves a preinitiation complex, and a S_2_ phospho CTD-code dictates that an enzyme becomes elongation competent [6]. In *T.brucei*, we found phosphorylated forms of ΨCTD within RNAP-II (Figure 2). Proteolytic digestion of phospho-ΨCTD produced what was most likely a heterogeneous set of differentially phosphorylated ΨCTDs. We note that there are ∼30 potential phosphorylation sites within the 2/3 ΨCTD that are absent from the 1/3 ΨCTD. Thus, we speculate that a phosphorylation-code occurs within the ΨCTD in *T. brucei* that may account for the two different RNAP-II-dependent reactions, namely, transcription of short, capped SL RNAs and long pre-mRNAs. Such a ΨCTD code may allow RNAP-II to specifically function at the SL RNA gene promoter, much like the S_7_ phosphorylation mark on the mammalian CTD directs polymerase specifically during small nuclear RNA transcription [4].

The structure of the canonical CTD is divided into three subdomains: the linker region, the central reiterated region that harbors intact and degenerate heptapeptide repeats, and the terminal short acidic peptide [23]. The ΨCTD parallels the CTD in structure; it contains a linker region of size similar to CTD, a central serine/proline-rich region, and a terminal short acidic peptide (Figure 1). In the case of 1/3 ΨCTD, the RNAP-II contained a ΨCTD having only the linker region plus a small part of the central serine/proline-rich region. This enzyme failed to rescue cell growth when wild-type RNAP-II was depleted. In addition, we did not observe phosphorylation of the 1/3 ΨCTD. In contrast, 2/3 ΨCTD of RNAP-II contained the linker region plus half of the central serine/proline-rich region. This enzyme was phosphorylated within the ΨCTD and allowed cell growth in the absence of wild-type RNAP-II. Taken together, it appears that the central serine/proline-rich region of the ΨCTD is important for polymerase function.

Although 2/3 ΨCTD could sustain cell growth, it generated increased levels of abortive transcripts. Nascent transcription in cells containing the 1/3 ΨCTD generated even higher levels of abortive transcripts. These transcripts were confirmed to be abortively initiated RNA, as they possessed 5′ tri-phosphate ends, a signature of transcription initiation (Figure 7B, C and D). Studies of the mammalian CTD indicate that an RNAP-II having only 5 heptapeptide repeats can bind a gene promoter but is defective in initiating transcription [24]. This mutant CTD is structurally similar to our 1/3 ΨCTD, because it contains the linker region, a very small part of the central region, and the terminal acidic region. Thus, the central region of both the ΨCTD and CTD plays a role in the transition from an initiating polymerase, which generates abortive RNAs, to an elongating enzyme, which produces full- length mRNAs.

An unexpected and interesting observation was made when an RNAP-II with 1/3 ΨCTD was expressed in cells along with wild-type levels of endogenous RNAP-II. Within 16 hours of 1/3 ΨCTD expression, cells ceased to move, changed their shape, formed large aggregates, and soon died (Figure 4A). These dramatic phenotypic changes were not observed when 1/3 ΨCTD was expressed in cells along with very low levels of endogenous RNAP-II. In this case cells merely stopped dividing by day 2 and slowly died (Figure 5A). This differential response may indicate that a wild-type RNAP-II, is required to synthesize protein-coding RNAs used in a stress response [25]. A stress response may account for the observed, abrupt morphological alterations in the cells expressing both the mutant and the endogenous polymerase. An actively transcribing polymerase in cells with abundant wild-type RNAP-II enables cells to respond to the stress imposed by the mutant ΨCTD. In contrast, cells with limited number of active polymerase could not. Indeed, *Drosophila* stress responses require transcription of heat shock proteins within minutes of the initial stress [26]. This rapid response is accompanied by the release of RNAP-II from its transcriptionally paused state on heat shock genes and depends on transcription elongation factors [27]. If a mechanism similar to this type of elongation control exists in trypanosomes, it raises the intriguing possibility of RNAP-II transcriptional control, heretofore unrecognized, in these organisms.

## Materials and Methods

### Plasmid constructs

The RNAi construct was made in p2T7-177 by inserting a 260 bp region of the RPB1 3′UTR at *Bam*HI and *Xho*I sites [28]. This plasmid integrates in the highly repressed 177 bp satellite region of a mini-chromosome. The construct was linearized with *Not*I, transfected into procyclic *T. brucei* cells and transgenic clonal cell lines selected by limiting dilution in the presence of phleomycin [29].

pAD71, designed to express a transgene constitutively, was derived from pUC19 and contains sequences for homologous recombination into the procyclin/GPEET locus, following linearization by *Not*I digestion. The transgene uses the endogenous procyclin/GPEET promoter and a 3′UTR derived from the aldolase mRNA. A blasticidin–resistance gene, which follows the transgene, is co-expressed using actin 5′ SAS and 3′UTR regions. pAD74 was derived from pLEW100, minus the luciferase open reading frame and containing a blasticidin-resistance gene in place of the phleomycin-resistance gene and an rRNA promoter in place of the procyclin/GPEET promoter.

Plasmid RPB3-SBP was generated by joining the RPB3 open reading frame (Tb927.3.550) with the protein A open reading frame and the Streptavidin binding protein (SBP) open reading frame (a gift from Larry Simpson) and cloned into pAD71 downstream from the procyclin/GPEET 5′UTR. To establish mutant RNAP-II, we generated epitope tagged versions of RPB1, with varying truncations of ΨCTD. A Ty1-tag (EVHTNQDPLD) [30] at the N-terminus allowed us to monitor the expression of mutant RPB1 proteins. Transgenes expressing mutant RNAP-II were designed with a well-characterized 3′UTR of aldolase (ALD) mRNA and thus resistant to the RNAi-targeting of endogenous RPB1 mRNA. The C-terminal five conserved amino acids, which contribute to RPB1 stability [24], was included in each of the four transgene constructs (Figure 1A). For constitutive expression of the transgenes, the tagged and mutant RPB1 ORFs were cloned into pAD71. For inducible expression of the transgenes, they were cloned into pAD74. All plasmid constructs were confirmed by DNA sequencing.

### Culturing and transfection of parasites

Procyclic (tsetse midgut form) wild-type *T. brucei* Lister 427 was grown in SDM-79 supplemented with 10% FCS as described [29]. For all transgene experiments, cells were selected and grown in medium containing tetracycline-free serum. The 29–13 cell line contains the T7 RNAP and the tetracycline repressor that are maintained by their co-expression with G418 and hygromycin-resistance genes [29]. p2T7-177 derivatives were transfected into 29–13 and stable cell lines were selected using 2.5 µg/ml phleomycin. pAD71 and pAD74 derivatives were transfected into 29–13 and selected using 10 µg/ml blasticidin. Inducible RNAi and transgene expression were regulated by addition of 500 ng/ml tetracycline. For metabolic labeling, 5×10^8^cells were incubated with 500 µCi of ^32^P-orthophosphate (9000 Ci/mmole) in 5 mls of phosphate-free DMEM (40 min, 26°C).

### Preparation of T. brucei nuclear extracts and protein purification

Nuclear extracts were made from the RPB3-SBP cells as previously described [31]. Protein was purified using tandem affinity chromatography; IgG-bound protein was washed and released from the matrix using TEV-protease. Eluted protein was bound to Streptavidin-agarose, washed and eluted with 2 mM biotin-containing buffer. For velocity sedimentation, nuclear extracts were loaded onto a 10-30% sucrose gradient as described [31]. Fractions were collected from the bottom of the tube and analyzed by immunoblotting. ^32^P-containing RPB1 was in-gel digested with endoproteinase Lys-C (Roche) as recommended by the manufacturer.

### Antibodies and western analyses

Polyclonal anti-ΨCTD antibody, produced in rabbits, recognizes endogenous RPB1 as well as the full-length, 2/3, and 1/3 ΨCTD RPB1 derived from transgenes. All four transgene-derived tagged-RPB1 proteins are also detected with monoclonal anti-Ty1 antibody, generated in the Gull laboratory and kindly provided by the Cross laboratory [30]. Anti-RPB4 antibody, produced in rabbits from recombinant protein as antigen, was used for immunoprecipitation experiments. For immunoprecipitations, nuclear extracts were incubated with anti-RPB4 antibody bound to protein A-sepharose, washed in buffer (20 mM Hepes [pH 7.9], 150 mM KCL, 150 sucrose, 2.5 mM MgCl_2_, 1 mM EDTA, 2.5 mM DTT, 0.1%NP-40, 1 µM each of pepstatin A, leupeptin, and PMSF) and analyzed by immunoblotting using ECL™ kit from Amersham.

### RNA analysis

Total cellular RNA from wild-type and transgenic cells was prepared using Trizol™ reagents. For Northern analysis, 5 µg of total RNA was separated on a formaldehyde-1.2% agarose gel in MOPS buffer [32]. RNA was fragmented by alkali-treatment, transferred to nylon membranes and hybridized with ^32^P-labelled oligonucleotides. Steady-state SL and U2 RNA levels were determined by primer extension analysis, using ^32^P-labeled oligonucleotides complementary to these RNAs. Nascent RNA was synthesized in lysolecithin-permeabilized cells using ^32^P-UTP [33] and profiled on 7M urea 10% polyacrylamide gel. Short transcripts were extracted from the gel, purified according to [34] and resolved on a 7M urea 25% polyacrylamide gel. Tobacco acid pyrophosphatase (Epicentre) and T4 RNA ligase (New England Biolabs) were used according to manufactures' protocols. Data were captured on a Typhoon PhosphorImager™ and quantification was performed using ImageQuant™ software.

### Microscopy

An Olympus BX61 microscope with Nomarski DIC optics was used to visualize live cells.

### Protein alignments

Alignments of RPB1 from *T. brucei*, human (P24928; Nc_000017.9), and Saccharomyces cerevisiae (YDL140C) were performed using CLUSTALW [35]. Results were manually altered to optimize comparisons and were represented using GeneDoc (http://www.psc.edu/biomed/genedoc).

## Supporting Information

Figure S1 Expression of RPB1 proteins with either 1/3 ψCTD or delta- ψCTD inhibits T. brucei cellular growth. (A) Growth curves of transgenic cell lines constitutively expressing a tagged full-length ψCTD (black square), 2/3 ψCTD (gray square), 1/3 ψCTD (grey circle) and delta ψCTD (black circle) RPB1. (B) Western analysis of whole cell lysates demonstrates expression of tagged RPB1s. Phosphorylated and non-phosphorylated forms of RPB1 are present. Anti-ψCTD antibody (top panel) detects the tagged version of the full ψCTD and 2/3 ψCTD, along with endogenous RPB1. Anti-tag (Ty1) BB2 antibody (bottom panel) detects only tagged-RPB1. Whereas the phosphorylated and non-phosphorylated forms of full ψCTD or 2/3 ψCTD are visible, only the non-phosphorylated forms of 1/3 ψCTD or delta- ψCTD are detected.
(0.39 MB PDF)

## References

[1] Cramer P, Armache KJ, Baumli S, Benkert S, Brueckner F (2008). Structure of eukaryotic RNA polymerases.. Annu Rev Biophys.

[2] Allison LA, Moyle M, Shales M, Ingles CJ (1985). Extensive homology among the largest subunits of eukaryotic and prokaryotic RNA polymerases.. Cell.

[3] Corden JL, Cadena DL, Ahearn JM, Dahmus ME (1985). A unique structure at the carboxyl terminus of the largest subunit of eukaryotic RNA polymerase II.. Proc Natl Acad Sci U S A.

[4] Egloff S, Murphy S (2008). Cracking the RNA polymerase II CTD code.. Trends Genet.

[5] Buratowski S (2003). The CTD code.. Nat Struct Biol.

[6] Phatnani HP, Greenleaf AL (2006). Phosphorylation and functions of the RNA polymerase II CTD.. Genes Dev.

[7] Palancade B, Bensaude O (2003). Investigating RNA polymerase II carboxyl-terminal domain (CTD) phosphorylation.. Eur J Biochem.

[8] Sims RJ, Belotserkovskaya R, Reinberg D (2004). Elongation by RNA polymerase II: the short and long of it.. Genes Dev.

[9] Meinhart A, Kamenski T, Hoeppner S, Baumli S, Cramer P (2005). A structural perspective of CTD function.. Genes Dev.

[10] Stiller JW, Duffield EC, Hall BD (1998). Amitochondriate amoebae and the evolution of DNA-dependent RNA polymerase II.. Proc Natl Acad Sci U S A.

[11] Dacks JB, Marinets A, Ford Doolittle W, Cavalier-Smith T, Logsdon JM (2002). Analyses of RNA Polymerase II genes from free-living protists: phylogeny, long branch attraction, and the eukaryotic big bang.. Mol Biol Evol.

[12] Schumacher MA, Lau AO, Johnson PJ (2003). Structural basis of core promoter recognition in a primitive eukaryote.. Cell.

[13] Guo Z, Stiller JW (2004). Comparative genomics of cyclin-dependent kinases suggest co-evolution of the RNAP II C-terminal domain and CTD-directed CDKs.. BMC Genomics.

[14] Gilinger G, Bellofatto V (2001). Trypanosome spliced leader RNA genes contain the first identified RNA polymerase II gene promoter in these organisms.. Nucl Acids Res.

[15] Smith JL, Levin JR, Ingles CJ, Agabian N (1989). In trypanosomes the homolog of the largest subunit of RNA Polymerase II Is encoded by two genes and has a highly unusual C-terminal domain structure.. Cell.

[16] Grondal EJM, Evers R, Kosubek K, Cornelissen AWCA (1989). Characterization of the RNA polymerases of Trypanosoma brucei: trypanosomal mRNAs are composed of transcripts derived from both RNA polymerase II and III.. EMBO J.

[17] Yurchenko VY, Lukes J, Jirku M, Zeledon R, Maslov DA (2006). Leptomonas costaricensis sp. n. (Kinetoplastea: Trypanosomatidae), a member of the novel phylogenetic group of insect trypanosomatids closely related to the genus Leishmania.. Parasitology.

[18] Patrick KL, Luz PM, Ruan JP, Shi H, Ullu E (2008). Genomic rearrangements and transcriptional analysis of the spliced leader-associated retrotransposon in RNA interference-deficient Trypanosoma brucei.. Mol Microbiol.

[19] Thomas S, Green A, Sturm NR, Campbell DA, Myler PJ (2009). Histone acetylations mark origins of polycistronic transcription in Leishmania major.. BMC Genomics.

[20] Chapman AB, Agabian N (1994). *Trypanosoma brucei* RNA Polymerase II Is Phosphorylated in the Absence of Carboxyl-Terminal Domain Heptapeptide Repeats.. J Biol Chem.

[21] Das A, Li H, Liu T, Bellofatto V (2006). Biochemical characterization of Trypanosoma brucei RNA polymerase II.. Mol Biochem Parasitol.

[22] Devaux S, Lecordier L, Uzureau P, Walgraffe D, Dierick JF (2006). Characterization of RNA polymerase II subunits of Trypanosoma brucei.. Mol Biochem Parasitol.

[23] Chapman RD, Palancade B, Lang A, Bensaude O, Eick D (2004). The last CTD repeat of the mammalian RNA polymerase II large subunit is important for its stability.. Nucleic Acids Res.

[24] Chapman RD, Conrad M, Eick D (2005). Role of the mammalian RNA polymerase II C-terminal domain (CTD) nonconsensus repeats in CTD stability and cell proliferation.. Mol Cell Biol.

[25] Lustig Y, Sheiner L, Vagima Y, Goldshmidt H, Das A (2007). Spliced-leader RNA silencing: a novel stress-induced mechanism in Trypanosoma brucei.. EMBO Rep.

[26] Saunders A, Core LJ, Lis JT (2006). Breaking barriers to transcription elongation.. Nat Rev Mol Cell Biol.

[27] Smith ER, Winter B, Eissenberg JC, Shilatifard A (2008). Regulation of the transcriptional activity of poised RNA polymerase II by the elongation factor ELL.. Proc Natl Acad Sci U S A.

[28] Wickstead B, Ersfeld K, Gull K (2002). Targeting of a tetracycline-inducible expression system to the transcriptionally silent minichromosomes of Trypanosoma brucei.. Mol Biochem Parasitol.

[29] Wirtz E, Leal S, Ochatt C, Cross GA (1999). A tightly regulated inducible expression system for conditional gene knock-outs and dominant-negative genetics in *Trypanosoma brucei*.. Mol Biochem Parasitol.

[30] Bastin P, Bagherzadeh Z, Matthews KR, Gull K (1996). A novel epitope tag system to study protein targeting and organelle biogenesis in *Trypanosoma brucei*.. Mol Biochem Parasitol.

[31] Das A, Zhang Q, Palenchar JB, Chatterjee B, Cross GAM (2005). Trypanosomal TBP functions with the multisubunit transcription factor tSNAP to direct Spliced leader RNA gene expression.. Mol Cell Biol.

[32] Sambrook J, Russell DW (2001). Molecular Cloning..

[33] Ullu E, Tschudi C (1990). Permeable trypanosome cells as a model system for transcription and trans-splicing.. Nucleic Acids Res.

[34] Bangs JD, Crain PF, Hashizume T, McCloskey JA, Boothroyd JC (1992). Mass spectrometry of mRNA cap 4 from trypanosomatids reveals two novel nucleosides.. J Biol Chem.

[35] Thompson JD, Higgins DG, Gibson TJ (1994). CLUSTAL W: improving the sensitivity of progressive multiple sequence alignment through sequence weighting, position-specific gap penalties and weight matrix choice.. Nucleic Acids Res.

