# Supplementary figures and images for: The Non-Canonical CTD of RNAP-II Is Essential for Productive RNA Synthesis in Trypanosoma brucei
Source: PLoS One. 2009 Sep 9;4(9):e6959. doi: 10.1371/journal.pone.0006959 (PMC2734056; doi:10.1371/journal.pone.0006959)

**A**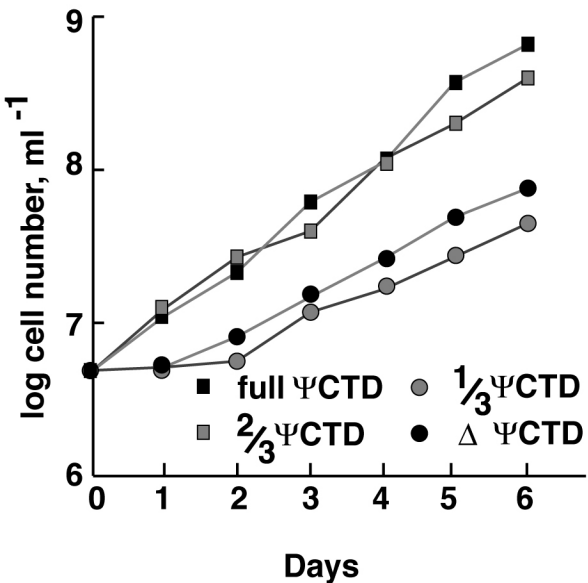**B**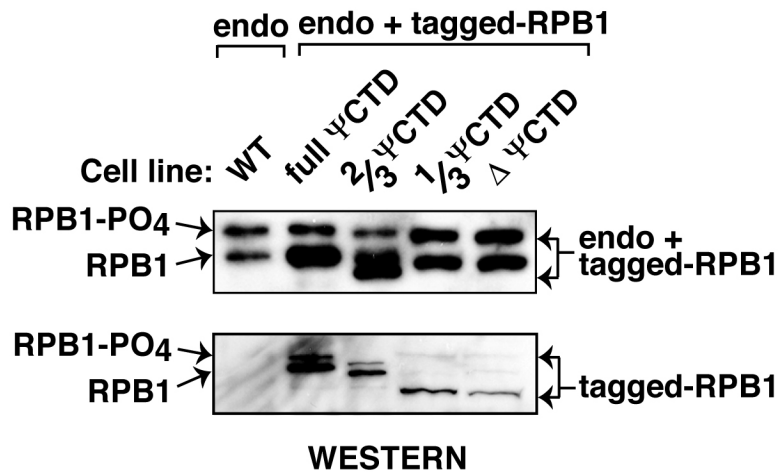

Figure S1.

Supplement: Figure S1 — Expression of RPB1 proteins with either 1/3 ψCTD or delta- ψCTD inhibits T. brucei cellular growth. (A) Growth curves of transgenic cell lines constitutively expressing a tagged full-length ψCTD (black square), 2/3 ψCTD (gray square), 1/3 ψCTD (grey circle) and delta ψCTD (black circle) RPB1. (B) Western analysis of whole cell lysates demonstrates expression of tagged RPB1s. Phosphorylated and non-phosphorylated forms of RPB1 are present. Anti-ψCTD antibody (top panel) detects the tagged version of the full ψCTD and 2/3 ψCTD, along with endogenous RPB1. Anti-tag (Ty1) BB2 antibody (bottom panel) detects only tagged-RPB1. Whereas the phosphorylated and non-phosphorylated forms of full ψCTD or 2/3 ψCTD are visible, only the non-phosphorylated forms of 1/3 ψCTD or delta- ψCTD are detected. (0.39 MB PDF) [file pone.0006959.s001.pdf]
